# Diffusion Tensor Imaging Studies on Arcuate Fasciculus in Stroke Patients: A Review

**DOI:** 10.3389/fnhum.2013.00749

**Published:** 2013-11-01

**Authors:** Sung Ho Jang

**Affiliations:** ^1^Department of Physical Medicine and Rehabilitation, College of Medicine, Yeungnam University, Taegu, South Korea

**Keywords:** arcute fasciculus, diffusion tensor imaging, stroke, aphasia

## Abstract

Aphasia is one of the most common and devastating sequelae of stroke. The arcuate fasciculus (AF), an important neural tract for language function, connects Broca’s and Wernicke’s areas. In this review article, previous diffusion tensor imaging (DTI) studies on the AF in stroke patients were reviewed with regard to the usefulness for diagnosis (seven studies), prediction of prognosis (two studies), and recovery of aphasia (three studies). Although scant studies on this topic have been conducted in stroke patients, DTI for the AF appears to provide useful information on the presence or severity of injury of the AF, prognosis prediction of aphasia, and recovery mechanisms of aphasia in stroke patients. Therefore, further DTI studies on these topics should be encouraged, especially studies on prognosis prediction and recovery mechanisms of aphasia. In addition, research on other neural tracts known to be involved in aphasia as well as the AF in both hemispheres should be encouraged.

## Introduction

Aphasia is a disorder of ability to communicate verbally resulting from brain injury (Geschwind, 1970; Schlaug et al., 2009). Aphasia is one of the most common and devastating sequelae of stroke. Approximately 24–38% of stroke patients have been reported to suffer from aphasia during acute stage (Wade et al., 1986; Pedersen et al., 1995; Laska et al., 2001; Engelter et al., 2006). A portion of these patients with aphasia show some degree of spontaneous recovery; this improvement of aphasia is observed mainly during the first 3 months after onset of stroke (Pedersen et al., 1995; Laska et al., 2001). As a result, more than 10% of stroke patients are known to suffer from aphasia at chronic stage (Pedersen et al., 1995; Robey, 1998; Berthier, 2005).

The arcuate fasciculus (AF) is the neural tract connecting Broca’s and Wernicke’s areas. Since identification of the AF, it has been regarded as an important neural tract for language along with the superior longitudinal fasciculus (SLF), inferior longitudinal fasciculus (ILF), inferior fronto-occipital fasciculus (IFOF), uncinate fasciculus (UF), and so on (Geschwind, 1970; Catani et al., 2005; Catani and Mesulam, 2008; Saur et al., 2008; Makris and Pandya, 2009; Weiller et al., 2011; Axer et al., 2012; Dick and Tremblay, 2012). Injury of the AF has been reported to cause various types of language problems such as deficits of comprehension or speech production as well as conduction aphasia (Anderson et al., 1999; Breier et al., 2008; Catani and Mesulam, 2008; Bernal and Ardila, 2009; Kim et al., 2011; Marchina et al., 2011). Therefore, clarification of the state of the AF in patients with aphasia would be important because it would be helpful to clinicians in estimating the neural state for aphasia, setting scientific rehabilitative strategies, and predicting prognosis of aphasia.

Previous studies on the AF have been conducted using intra-operative mapping techniques and conventional brain imaging techniques such as CT or MRI (Benson et al., 1973; Duffau et al., 2002). As a result, because the AF cannot be clearly discriminated from adjacent structures, accurate evaluation of the AF has been difficult. Diffusion tensor imaging (DTI) allows for evaluation of white matter tracts by virtue of its ability to visualize water diffusion characteristics (Basser and Pierpaoli, 1996; Mori et al., 1999). In normal white matter, water molecules move with relative freedom in a direction parallel to nerve fiber tracts; however, their movements, are restricted across the tracts, causing diffusion anisotropy of the white matter. Diffusion anisotropy has been used in evaluation of the extent of fiber injury or recovery in pathologies that affect white matter (Basser and Pierpaoli, 1996; Mori et al., 1999). Diffusion tensor tractography (DTT), derived from DTI, allows for three-dimensional reconstruction and estimation of the AF (Catani et al., 2005; Nucifora et al., 2005; Glasser and Rilling, 2008). Therefore, DTI has been employed in many studies for evaluation of the AF in various brain pathologies, including stroke (Yamada et al., 2007; Breier et al., 2008, 2011; Hosomi et al., 2009; Rauschecker et al., 2009; Schlaug et al., 2009; Zhang et al., 2010; Kim et al., 2011; Kwon and Jang, 2011; Marchina et al., 2011; Song et al., 2011; Wilson et al., 2011; Kim and Jang, 2013; Kummerer et al., 2013; Yeatman and Feldman, 2013).

In this review, DTI studies on the AF in stroke patients were reviewed. Relevant studies were identified using the following electronic databases (Pubmed and MEDLINE) from 1966 to 2013. The following key words were used: DTI, DTT, AF, aphasia, and stroke. This review was limited to studies on the AF in human stroke patients. Finally, 12 studies were selected and reviewed with respect to diagnosis, recovery, and prognosis prediction of aphasia in stroke patients (Yamada et al., 2007; Breier et al., 2008, 2011; Hosomi et al., 2009; Schlaug et al., 2009; Zhang et al., 2010; Kim et al., 2011; Kwon and Jang, 2011; Marchina et al., 2011; Song et al., 2011; Kim and Jang, 2013; Kummerer et al., 2013).

## Neural Tracts Which are Involved in Language Function

**Figure 1:**
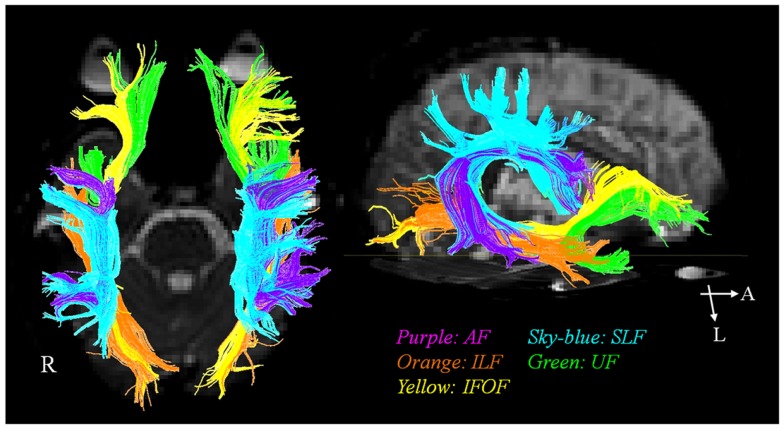
**The diffusion tensor tractographies of neural tracts for language: the arcuate fasciculus (AF), the superior longitudinal fasciculus (SLF), the inferior longitudinal fasciculus (ILF), the uncinate fasciculus (UF), and inferior fronto-occipital fasciculus (IFOF)**.

In the past, the AF has been regarded as a unique neural tract connecting Broca’s and Wernique’s areas (Geschwind, 1970). More recently, involvement of various other neural tracts in language function has also been reported (Geschwind, 1970; Catani et al., 2005; Catani and Mesulam, 2008; Duffau, 2008; Saur et al., 2008; Makris and Pandya, 2009; Weiller et al., 2011; Axer et al., 2012; Dick and Tremblay, 2012; Kummerer et al., 2013). These neural tracts for language have recently been classified according to two categories: the dorsal stream for phonation and ventral stream for comprehension. The dorsal stream comprises the AF and SLF, in contrast, the ventral stream includes the ILF, IFOF, UF, and extreme capsule (Figure 1). The dorsal stream connects temporoparietal with frontal premotor regions through the AF and SLF, and integrates sensorimotor processing, e.g., in repetition of speech (Geschwind, 1970; Catani et al., 2005; Catani and Mesulam, 2008; Duffau, 2008; Saur et al., 2008; Makris and Pandya, 2009; Weiller et al., 2011; Axer et al., 2012; Dick and Tremblay, 2012; Kummerer et al., 2013). The ventral stream connects temporal and prefrontal regions via the extreme capsule and mediates meaning, e.g., in auditory comprehension (Geschwind, 1970; Catani et al., 2005; Catani and Mesulam, 2008; Duffau, 2008; Saur et al., 2008; Makris and Pandya, 2009; Weiller et al., 2011; Axer et al., 2012; Dick and Tremblay, 2012; Kummerer et al., 2013). The AF is the major fiber tract of the dorsal stream and the IFOF is the major tract of the ventral stream (Axer et al., 2012).

## Anatomy of the Arcuate Fasciculus

In the dorsal stream of the language network, the AF appears to be primarily involved in the phonology pathway, in contrast, the SLF III appears to be involved in speech perception and articulatory pathways (Catani et al., 2005; Duffau, 2008; Saur et al., 2008; Axer et al., 2012). The AF is known to originate from Brodmann’s Areas (BA) 22, 21, and 37, and terminates in BA 44, 45, and 6 (Hong et al., 2009; Axer et al., 2012). However, Bernal and Altman (2008) studied 12 normal right-handed subjects and found that the AF projection to Broca’s areas was absent in 10 subjects (83.3%), and present in 2 (16.6%). By contrast, the AF’s rostral endpoints were found in the precentral gyrus in 100% of subjects (Bernal and Altman, 2008; Bernal and Ardila, 2009). These findings suggest the possibility that the language relay station is located in the precentral gyrus (BA 6; an area with premotor function), which is connected to Broca’s area and BA 4 (Bernal and Ardila, 2009).

Catani et al. (2005) reported on the perisylvian language network, in which there were two connection pathways: the classical direct long segment connecting Wernicke’s and Broca’s areas, and the indirect pathway consisting of two segments: anterior segment linking Broca’s territory with Geschwind’s territory (inferior parietal lobule) and the posterior segment linking Geschwind’s territory with Wernicke’s territory. Findings of this study indicate that the functions of these two pathways would differ; the direct pathway appears to be related to phonologically based language function, such as repetition, and the indirect pathway is related to semantically based language function.

Glasser and Rilling (2008) demonstrated that the AF was divided into two segments terminating in the posterior superior temporal gyrus (BA 22), which was connected to BA 44 and 6, and in the middle temporal gyrus (BA 21 and 37), which was connected to BA 44, 45, 6, and 9. Based on the findings of previous studies, they suggested that the functions of these two segments would differ; the segment to the superior temporal gyrus plays a role in repetition (phonological content), while the segment to the middle temporal gyrus functions in spontaneous speech (lexical-semantic content).

Several studies have suggested that the AF transmits information not only from the temporal to frontal areas, but also in the opposite direction (Bullmore et al., 2000; Matsumoto et al., 2004; Bernal and Ardila, 2009). This finding suggests that the role of the AF may be more complex than simply transmitting information between Wernicke’s and Broca’s areas (Bernal and Ardila, 2009). Bidirectional transfer of information may indicate that information regarding language production is important for language understanding (Bernal and Ardila, 2009).

## Advantages and Limitations of DTI as a Tool for Evaluation of the AF in Stroke Patients

The configurations and three parameters [fractional anisotropy (FA), apparent diffusion coefficient (ADC), and fiber number] of DTI for the AF have been used in DTI studies on the AF in stroke patients (Yamada et al., 2007; Breier et al., 2008, 2011; Hosomi et al., 2009; Schlaug et al., 2009; Zhang et al., 2010; Kim et al., 2011; Kwon and Jang, 2011; Song et al., 2011; Kim and Jang, 2013). FA is the most widely used parameter of DTI (Mori et al., 1999; Assaf and Pasternak, 2008; Neil, 2008). The FA value represents the degree of directionality of microstructures, such as axons, myelin, and microtubules. The FA value can increase with increased organization of white matter tracts; in contrast, a decrease in the condition is related to disintegration of a neural tract. The ADC value indicates the magnitude of water diffusion, which can increase with some forms of pathology, particularly vasogenic edema or accumulation of cellular debris from axonal damage (Mori et al., 1999; Assaf and Pasternak, 2008; Neil, 2008; Kim and Jang, 2013). In addition, the configuration in terms of integrity and size of the AF on DTT has been used as a DTT parameter in stroke patients (Yamada et al., 2007; Zhang et al., 2010; Kim et al., 2011; Kwon and Jang, 2011; Kim and Jang, 2013; Kummerer et al., 2013).

Diffusion tensor imaging is known to have the following advantages for research on injury of the AF in stroke patients. First, DTI has unique value in its ability to diagnose hidden or ambiguous lesions that cannot be easily detected on conventional brain MRI (Yamada et al., 2007; Kim et al., 2011). Second, serial DTI scanning of the AF allows for estimation of changes of an injured AF, for example, regeneration, degeneration, or resolution of peri-AF edema of an injured AF (Schlaug et al., 2009). Third, asymmetry of the AF between the right and left hemispheres on DTT can provide another parameter for evaluation of an injured AF (Nucifora et al., 2005; Powell et al., 2006; Hosomi et al., 2009). Fourth, other neural tracts involved in language function can be analyzed along with the AF and compared with the state of the AF (Breier et al., 2008; Marchina et al., 2011; Kummerer et al., 2013). However, several limitations of DTI should be considered (Lee et al., 2005; Parker and Alexander, 2005; Yamada et al., 2009). First, the fiber tracking technique is operator-dependent. Therefore, results can be misleading (Lee et al., 2005). Second, DTI may underestimate or overestimate fiber tracts. The problem associated with kissing fiber in regions of fiber complexity can prevent full reflection of the underlying fiber architecture by DTI (Parker and Alexander, 2005; Yamada et al., 2009). Many recent studies have attempted to solve this limitation using probabilistic tractography (Parker and Alexander, 2005). Third, DTT cannot determine the exact cortical origin and termination of fibers; therefore, we can only define the territories of fiber projection (Catani et al., 2005). Studies using a combination of functional neuroimaging techniques can compensate for this limitation of DTI.

## DTI Studies on the Arcuate Fasciculus in Stroke Patients

Since introduction of DTI, 12 studies have reported on the AF in stroke patients with aphasia (Yamada et al., 2007; Breier et al., 2008, 2011; Hosomi et al., 2009; Schlaug et al., 2009; Zhang et al., 2010; Kim et al., 2011; Kwon and Jang, 2011; Marchina et al., 2011; Song et al., 2011; Kim and Jang, 2013; Kummerer et al., 2013). We classified these studies with regard to diagnosis, prognosis prediction, and recovery of aphasia (Table 1).

**Table 1:** **Previous diffusion tensor imaging studies on the arcuate fasciculus in stroke patients**.

Reference	Patient no.	Stroke type	Post-stroke duration to DTI	Clinical evaluation method	Paramaters of DTI	Analyzed other neural tracts
**DIAGNOSIS**						
Yamada et al. (2007)	1	Cerebral infarct	3 days		FA configuration (size)	
Breier et al. (2008)	20	16: MCA region (15: ischemia 1: hemorrhage) 4: subcortical region (1: ischemia 1: hemorrhage)	1 month < (1 ∼ 72)	WAB (AQ)	FA (single ROI)	SLF, UF
Zhang et al. (2010)	10	Cerebral infarct (conduction aphasia)			FA single (ROI) configuration (morphology)	
Marchina et al. (2011)	30	MCA region	11 months < (mean 35)	Boston naming test, Boston diagnostic aphasia evaluation	Tract-lesion overlap volume	Extreme capsule, UF
Song et al. (2011)	10	Cerebral infarct (conduction aphasia)		WAB	FA (single ROI, DTT)	
Kim et al. (2011)	5	Legion at around left AF (two: ischemia three: hemorrhage)	Mean 34 days (13 ∼ 106)	WAB	FA (DTT), fiber number (DTT), configuration (integrity)	
Kummerer et al. (2013)	100	Embolic stroke of left hemiplegia	Mean 3 days		Voxel wise lesion-behavior mapping analysis	Dorsal and ventral white matter tracts
**PROGNOSIS**						
Hosomi et al. (2009)	13	Left MCA infarct	48 h ≥ (3 ∼ 44)	NIH stroke scale, (language score)	FA (DTT), Fiber number	
Kim and Jang (2013)	25	Left basal ganglia and corona radiata	DTI: 19.6 days, (9 ∼ 30), one 30 days ≥ (20.6 days), two 3 months < (171.5 days)	WAB (AQ)	FA (DTT), ADC (DTT), configuration (integrity)	
**RECOVERY**						
Schlaug et al. (2009)	6	Left hemisphere stroke	1 year <	No. of correct information units during spontaneous speech	Fiber number volume	
Kwon and Jang (2011)	1	Cerebral infarct		WAB	Configuration (integrity)	
Breier et al. (2011)	1	Left MCA infarct	5 years	WAB	FA	

*DTI, diffusion tensor imaging; MCA, middle cerebral artery; FA, fractional anisotropy; WAB, western apahasia battery; AQ, aphasia quotient; ROI, region of interest; SLF, superior longitudinal fasciculus; UF, uncinate fasciculus; DTT, diffusion tensor tractography; AF, arcuate fasciculus; ADC, apparent diffusion coefficient*.

### Diagnosis

To the best of our knowledge, seven studies have reported on the usefulness of the AF for diagnosis of aphasia in stroke patients. Yamada et al. (2007) reported on a patient who showed conduction aphasia following a corona radiata infarct. The patient showed severely impaired repetition with paraphasic errors. Although no lesion was observed in Broca’s or Wernicke’s areas on diffusion-weighted images of the brain, partial injury of the left AF in terms of the FA value and configuration of the AF was demonstrated on DTT: decreased FA value and smaller size of the left AF, compared with the right AF. During the next year, Breier et al. (2008) recruited 20 patients (16 middle cerebral artery territory stroke lesions: 15 ischemia, one hemorrhage; four subcortical regions: one ischemia, one hemorrhage) who were at least 1 month post-stroke onset (mean 22 months, 1–72 months) (Breier et al., 2008). They measured FA value in the left AF, SLF, and UF using a single region of interest (ROI) method. Lower FA values of the AF and SLF of the left hemisphere were found to show correlation with decreased ability of repetition, which was measured using aphasia quotient (AQ) of Western Aphasia Battery (WAB). Comprehension deficits on comprehension AQ after stroke also showed an association with lower FA value of the left AF. Consequently, findings of this study demonstrated that injuries of the AF and SLF in the left hemisphere could cause conduction aphasia in stroke patients. Subsequently, Zhang et al. (2010) recruited 10 patients who showed conduction aphasia after a cerebral infarct. They reported a decrease in FA value, which was measured using the single ROI method and different configuration of the left AF in 10 patients with conduction aphasia. Marchina et al. (2011) estimated the volume of three language-related neural tracts (the AF, extreme capsule, and UF) affected by a stroke lesion in 30 patients with stroke lesion in the middle cerebral artery territory who were at least 11 months post-stroke onset. They found that lesion load of the AF, irrespective of extreme capsule and UF, were predictive of language function in terms of rate, informativeness, efficacy of speech, and naming ability, which were measured using the Boston Naming Test and Boston Diagnostic Aphasia evaluation. During the same year, Song et al. (2011) recruited five patients with Broca-like conduction aphasia and five patients with Wernicke-like conduction aphasia. FA values of the left Broca’s area and the left anterior segment of the AF were smaller than those of the right side in patients with Broca-like conduction aphasia. By contrast, FA values of the left Wernicke’s area and the left posterior segment of the AF were smaller in patients with Wernicke-like conduction aphasia than in those of the right side. As a result, they demonstrated that a lesion involving Broca’s area and the anterior segments of the AF would lead to Broca-like conduction aphasia, whereas a lesion involving Wernicke’s area and posterior segments of the AF would lead to Wernicke-like conduction aphasia. Kim et al. (2011) investigated the clinical usefulness of DTT of the AF in five stroke patients who had lesions in the left corona radiata and basal ganglia level: one patient with mild dysarthria showed a normal left AF in terms of integrity and DTT parameters, one patient with conduction aphasia showed partial injury of the left AF, two patients with Broca’s aphasia who had no brain lesions at Broca’s area on conventional brain MRI, showed disruptions of the left AF over the stroke lesions, and one patient with global aphasia whose left AF was not reconstructed due to severe injury and Wallerian degeneration. Therefore, results of this study suggested that DTT for the AF could provide useful information on the presence or severity of injury of the AF, which could not be detected on conventional brain MRI in stroke patients. In addition, it could be helpful to some extent in classification of the aphasia type of stroke patients. Recently, Kummerer et al. (2013) investigated correlation of acute impairment of repetition and comprehension with lesions of the dorsal or ventral stream in 100 patients with acute embolic stroke (mean 3 days after onset) with aphasia, which was estimated using either the Aachen Aphasia Bedside test or the Aachen Aphasia test (Kummerer et al., 2013). They combined voxelwise lesion-behavior mapping with the dorsal and ventral white matter fibers tracts using probabilistic fiber tracking. According to their findings, repetition impairments were mainly associated with lesions located in the posterior temporoparietal region with a statistical lesion maximum in the periventricular white matter in projection of the dorsal SLF and AF. However, lesions associated with comprehension deficits were found more ventral-anterior in the temporal prefrontal region with a statistical lesion maximum between the insular cortex and the putamen in projection of the ventral extreme capsule. Individual lesion overlap with the dorsal fiber tract showed negative correlation with repetition, in contrast, lesion overlap with the ventral fiber tract showed negative correlation with comprehension.

### Prognosis

Prediction of prognosis is important in stroke patients because it could provide useful information for planning specific rehabilitation strategies and for estimating duration of rehabilitation (Kim and Jang, 2013). However, only a few studies on this topic have demonstrated the clinical usefulness of DTT for the AF (Hosomi et al., 2009; Kim and Jang, 2013).

Hosomi et al. (2009) recruited 13 patients with an infarct in the left middle cerebral artery territory who underwent DTI scanning within 48 h after stroke onset. According to the severity of aphasia at discharge using the language score of NIH Stroke Scale, the patients were assigned to two groups: six patients in the aphasic group and seven patients in the non-aphasic group. Loss of leftward asymmetry in fiber number of the AF predicted aphasia at discharge (13–52 days after onset) irrespective of FA value. As a result, findings of this study demonstrated that asymmetry of fiber number of the AF on DTT, which was assessed in acute stage of cerebral infarct, could predict prognosis of aphasia. Recently, Kim and Jang (2013) attempted to determine whether the integrity of the left AF could be a factor in prediction of prognosis of aphasia in 25 stroke patients with aphasia. The AQ of WAB was used for assessment of aphasia in the early stage of stroke (10–30 days) and at approximately 6 months after onset. At 6 month evaluation, the AQ values of patients whose left AF was disrupted (52.43 ± 25.75, full mark: 100) and patients whose left AF was preserved around the lesion (68.08 ± 15.76) were higher than that of patients whose AF was not reconstructed (10.98 ± 3.90). Therefore, findings of this study demonstrated that the aphasia outcome of patients whose left AF could be reconstructed was better than that in patients whose left AF could not be reconstructed, irrespective of preservation of integrity of the AF. This study suggested that evaluation of the left AF using DTT in the early stage of stroke could be helpful in predicting aphasia outcome in stroke patients.

### Recovery

According to previous studies on the recovery mechanism of aphasia, several recovery mechanisms of aphasia can be assumed as follows: contribution of either peri-lesional brain region in the affected hemisphere or homologous language regions in the unaffected hemisphere, or recovery via other neural tracts such as the SLF (Thulborn et al., 1999; Fernandez et al., 2004; Saur et al., 2006; Raboyeau et al., 2008; Bernal and Ardila, 2009). However, studies using DTI on the recovery mechanisms of aphasia in stroke have been fewer (Schlaug et al., 2009; Breier et al., 2011; Kwon and Jang, 2011).

Schlaug et al. (2009) reported that the fiber number and volume of the right AF were increased after 75–80 intonation-based speech therapy sessions in six chronic stroke patients with Broca’s aphasia. Findings of this study indicated that intense, long-term melodic intonation therapy could lead to remodeling of the right AF. Subsequently, Kwon and Jang (2011) reported on a patient who showed excellent recovery of aphasia despite complete injury of the AF due to a cerebral infarct in the left centrum semiovale and corona radiata (Kwon and Jang, 2011). This right-handed patient presented with severe global aphasia on WAB at 1 week after onset (AQ: 12). The patient underwent rehabilitative therapy, including speech therapy and medication, which is known to facilitate recovery from aphasia, for a period of 24 months (Bakheit, 2004; Tanaka, 2007). His aphasia had improved to a nearly normal state at 30 months after onset (AQ:93). The left AF showed a complete disruption on 27 month DTT. Findings of this study suggested the possibility that aphasia can show good recovery, despite disruption of the left AF. During the same year, Breier et al. (2011) reported the effect of Constraint Induced Language Therapy, which is a language therapy based on the principle of use-dependent learning (3 h sessions, 4 days a week and for 3 weeks for a total of 36 h of treatment, treatment task was a dual card task where each patient took turns either requesting a matching card from a semantic category from the other patient, or responding to that request) in a chronic patient with middle cerebral artery infarct (Breier et al., 2011). They found that the FA value of the left AF was increased immediately and 3 months after starting the therapy along with the improvement of AQ and repetition on WAB.

## Conclusion

In this review article, previous DTI studies on the AF in stroke patients were reviewed in terms of the usefulness for diagnosis, prediction of prognosis, and recovery of aphasia. Fewer DTI studies on this topic in stroke patients have been reported, compared with other functions, such as motor or cognitive functions. However, DTI for the AF appears to provide useful information on the presence or severity of injury of the AF, prognosis prediction of aphasia, and recovery mechanisms of aphasia in stroke patients. Therefore, conduct of further DTI studies on this topic should be encouraged, especially studies on prediction of prognosis and recovery mechanisms of aphasia. In addition, research on other neural tracts known to be involved in aphasia as well as the AF in the dominant hemisphere should be encouraged. Likewise, conduct of studies on changes of the neural tract, including the AF in the non-dominant hemisphere, is needed. On the other hand, studies using a combination of functional neuroimaging techniques would be helpful in compensating for the limitation of DTI at the cortex level.

## Conflict of Interest Statement

The author declares that the research was conducted in the absence of any commercial or financial relationships that could be construed as a potential conflict of interest.
